# Using Temporary Prostatic Stents to Eliminate Bacterial Colonization in Men with Chronic Indwelling Catheters: A Pilot Study

**DOI:** 10.7759/cureus.3152

**Published:** 2018-08-16

**Authors:** Sarat Sabharwal, Sahil Sabharwal

**Affiliations:** 1 Urology, Orlando VA Medical Center , Orlando, USA; 2 Biology, University of Central Florida, Orlando, USA

**Keywords:** bacterial colonization, temporary prostatic stents, tps, chronic indwelling catheters, va, veterans

## Abstract

Background

Chronic urinary catheterization is commonly associated with chronic bacterial colonization and high rates of symptomatic infection that increase morbidity and mortality. This study describes the results of replacing chronic catheters with temporary prostatic stents (TPS) to reduce bacterial colonization rates.

Methods

Twenty-two chronically catheterized adult male patients were enrolled. Upon removal, the indwelling urinary catheter (IUC) was cultured to identify the presence and type of bacterial flora. The IUC was replaced with a TPS. All patients had five consecutive TPS placed on a 30-day cycle. TPS cultures were obtained at removal of each TPS.

Results

All patients (100%) demonstrated bacterial colonization at baseline (removal of the IUC). After the third month with TPS, the colonization had fallen to 5% and remained at 5% for the final two months of TPS placement.

Conclusions

This pilot study suggests that replacing an IUC with a TPS interrupts the cycle of bacterial colonization in the urinary tract. This approach could be a strategy for eliminating multi-drug resistant organisms from the urinary tract of men with urinary retention.

## Introduction

The placement of an indwelling urinary catheter (IUC) often results in bacteriuria or bacterial colonization [1,2]. Bacterial colonization rates are estimated at approximately 5% per day of IUC use, with nearly 100% of patients colonized after 30 days of IUC use [3].

As of 2016, the VA Healthcare System has treated approximately 17 million male patients with a median age of 65 years [4]. Older male patients commonly have increased bladder outlet resistance due to benign prostate hyperplasia (BPH). These patients can suffer from urinary retention when the bladder fails to generate adequate pressure to overcome elevated outlet resistance. Due to comorbidities and other medical circumstances, these patients often require chronic catheterization to passively empty the bladder.

This study describes our experience replacing the IUC with a temporary prostatic stent (TPS) in male chronic retention patients with the goal of reducing bacterial colonization rates. Potential advantages of TPS include the resumption of the natural filling and emptying cycle of the bladder without device components that extend outside of the body. We postulate that by eliminating the IUC, the TPS will allow the body to naturally protect against bacterial colonization.

## Materials and methods

We retrospectively reviewed data from chronically catheterized male retention patients who had an IUC replaced with a TPS during the period from January 2015 to July 2017. All patients were required to be catheterized for more than 30 days due to chronic urinary retention associated with bladder outlet obstruction. The TPS was offered to these patients on a voluntary basis as an alternative to their IUC and all patients were treated in an outpatient setting.

At the initial evaluation, the IUC was removed and the IUC was cultured. The colonization data was recorded to be used as a baseline. A TPS (Spanner Temporary Prostatic Stent, SRS Medical, North Billerica, MA, USA) was placed immediately following the removal of the IUC. Each TPS was changed approximately monthly for five consecutive months. At each TPS removal, a TPS culture was obtained. Therefore, a total of six device cultures were obtained for each patient: the first culture at baseline (post IUC removal) and then again after each of the five TPS removals. A colonization result (single organism count > 10^5^ CFU/ml) did not result in antibiotic treatment nor a delay in TPS placement.

**Figure 1:**
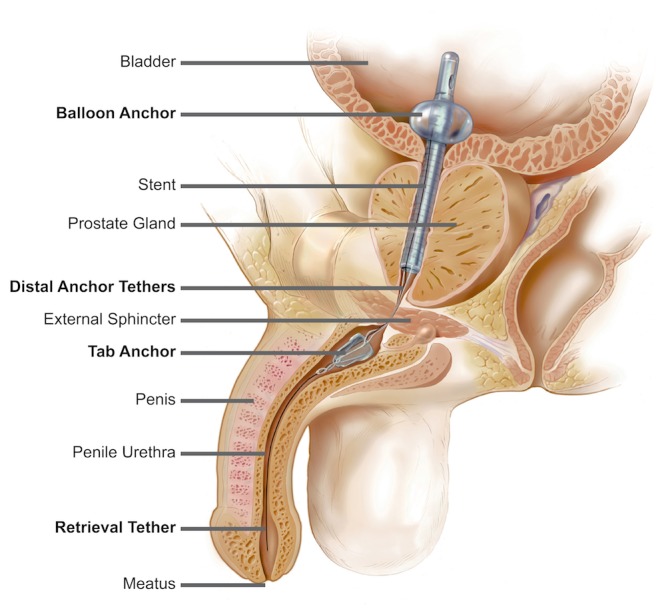
Sagittal Plane of the Male Urinary Tract with TPS in Place Courtesy of SRS Medical [5]. TPS - temporary prostatic stent

The TPS (Figure 1) is composed of a silicone tube that holds open the prostatic urethra, de-obstructing the bladder outlet while allowing the patient to restore his natural micturition cycle. The device is anchored at the bladder neck by a 5cc balloon and in the bulbar urethra with soft silicone distal anchor. Nylon tethers connect the stent body to the distal anchor, allowing the external sphincter to close and maintain continence.

The TPS is delivered on a device introducer and is inserted into the urethra with topical anesthesia. The TPS is placed in a procedure similar to the placement of an IUC, in which the proximal end of the TPS is inserted into the bladder, and its silicone balloon is inflated with 5 cc of sterile water. The device introducer is then removed, leaving the TPS in place. The TPS has an integral retrieval tether which is pulled to deflate the balloon at the time of device removal. The TPS comes in six different lengths, and prostatic urethral length is measured with a sizing device that is included with the TPS.

## Results

**Figure 2:**
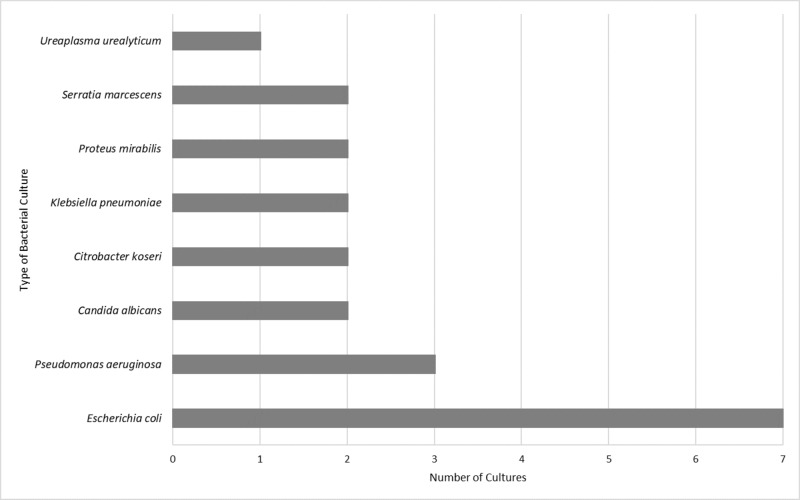
Microbial Profile at IUC Removal IUC: indwelling urinary catheter

Twenty-two male patients (age range 52-78) are included in this report. All patients (22 of 22) were colonized at the time of the initial IUC removal. Figure 2 shows the distribution of bacterial flora present at the time of initial IUC removal. The most common organism was *E. coli* (7 of 22).

**Figure 3:**
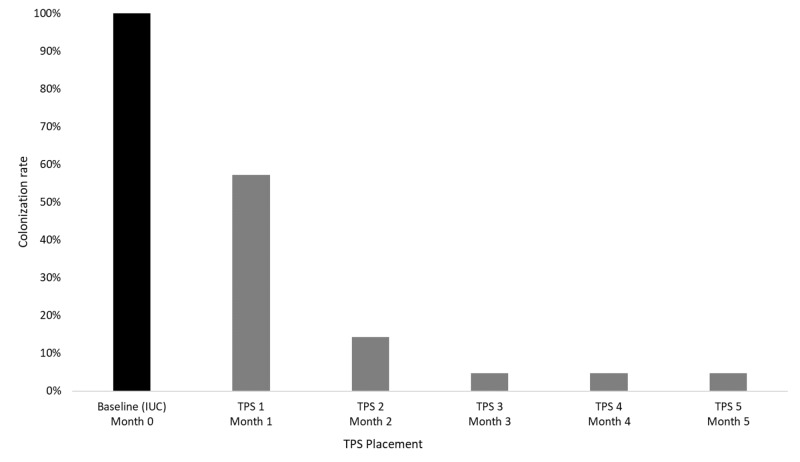
Colonization Rate at IUC and TPS Removals IUC: indwelling urinary catheter; TPS: temporary prostatic stents

All 22 patients received an initial TPS, and 21 of the 22 patients received all five consecutive TPS. The percentage of colonized patients gradually decreased over the initial TPS placements. After the first TPS placement, 13 (59%) remained colonized. After the second TPS placement, three remained colonized (14%). After the third, fourth, and fifth TPS placements, only one patient was colonized (5%). Figure 3 shows the colonization rate at each culture. Table 1 and Table 2 detail each patient’s culture results, including specific bacterial flora found if colonized.

**Table 1:** Detailed Culture Results Culture Results Key Free of colonization: *, *Escherichia coli*: 1, *Pseudomonas aeruginosa*: 2, *Candida albicans*: 3, *Citrobacter koseri*: 4, *Klebsiella pneumoniae*: 5, *Proteus mirabilis*: 6, *Serratia marcescens*: 7, *Ureaplasma urealyticum*: 8, Mixed flora: 9.

Subject ID	Baseline	Stent 1 removal	Stent 2 removal	Stent 3 removal	Stent 4 removal	Stent 5 removal
Subject 01	Colonized^1^	*	*	*	*	*
Subject 02	Colonized^1^	*	*	*	*	*
Subject 03	Colonized^4^	*	*	*	*	*
Subject 04	Colonized^5^	*	*	*	*	*
Subject 05	Colonized^1^	*	*	*	*	*
Subject 06	Colonized^1^	*	*	*	*	*
Subject 07	Colonized^1^	*	*	*	*	*
Subject 08	Colonized^1^	*	*	*	*	*
Subject 09	Colonized^5^	*	*		*	*
Subject 10	Colonized^3^	Colonized^3^	*	*	*	*
Subject 11	Colonized^1^	Colonized^1^	*	*	*	*
Subject 12	Colonized^8^	Colonized^1^	*	*	*	*
Subject 13	Colonized^4^	Colonized^1^	*	*	*	*
Subject 14	Colonized^1^	Colonized^1^				
Subject 15	Colonized^2^	Colonized^2^	*	*	*	*
Subject 16	Colonized^3^	Colonized^5^	*	*	*	*
Subject 17	Colonized^6^	Colonized^6^	*	*	*	*
Subject 18	Colonized^6^	Colonized^1^	*	*	Colonized^9^	*
Subject 19	Colonized^6^	Colonized^6^	*	Colonized^1^	*	*
Subject 20	Colonized^2^	Colonized^2^	Colonized^9^	*	*	*
Subject 21	Colonized^2^	Colonized^2^	Colonized^1^	*	*	*
Subject 22	Colonized^7^	Colonized^7^	Colonized^1^	*	*	Colonized^7^

**Table 2:** Summary of Culture Results

Description	Baseline	Stent 1 removal	Stent 2 removal	Stent 3 removal	Stent 4 removal	Stent 5 removal
Numbers of subjects cultured	22	22	21	20	21	21
Colonized subjects	22	13	3	1	1	1
Colonization rate	100%	59%	14%	5%	5%	5%

A total of 106 TPSs were utilized with the 22 patients. Two subjects have missing data, which was treated as missing for analysis: (i) subject 14 was lost to follow-up after the first TPS placement and therefore only had one TPS placement; (ii) the culture result of subject 9 after his third TPS removal is missing.

In addition, the dwell times for two TPS placements were extended to eight weeks due to patient scheduling. Of these two placements, one had colonization related to the longer dwell time of the TPS (subject 22, TPS placement 5).

Patients were asked about their experience with the TPS, particularly in comparison with their previous experience with IUC. All patients reported satisfaction with the TPS experience and there were no reported complications specific to the TPS.

## Discussion

Our study demonstrates a significant reduction in bacterial colonization by replacing a long-term IUC with serial placements of TPS. The TPS offers several potential benefits to lower risk of bacterial colonization, specifically: (a) the absence of external components, eliminating a potential pathway for external organisms to enter the urinary tract; (b) the absence of a large diameter tube in the penile urethra, allowing for the maintenance of the urethral mucosal coaptation as a natural bacterial barrier; and (c) the maintenance of filling and emptying of the bladder, allowing for the natural flushing mechanism to wash bacteria from the lower urinary tract.

We observed a gradual decrease in the colonization rate over the first three TPS placements. After the first TPS placement, 59% of patients remained colonized. After the second TPS placement, only 14% remained colonized, then settling at 5% for the remaining three TPS placements. We speculate that the gradual drop in colonization rates (59% of patients remained colonized after the first TPS placement) are the result of initially placing a clean TPS into a colonized bladder and a biofilm forming on the TPS. We believe that treating a colonized bladder prior to TPS treatment (such as an iodine bladder lavage) could accelerate the elimination of colonization, and should be investigated further.

Previous reports of the use of this TPS have also demonstrated low rates of bacteriuria [6-8]. Abdul-Muhsin et al. reported that no bacterial colonization was found in TPS placements with an indwell duration of fewer than 20 days [6]. Roach reported that with an average indwell duration of 30 days, symptomatic urinary tract infections (SUTI) occurred in six of 214 TPS placements (2.8%), resulting in an incident rate of 0.93 SUTI per 1,000 TPS days [8].

TPS has also been reported to have secondary benefits for patients. The absence of external components improves mobility and ambulation with no risk of meatal erosion or injury. In addition, quality of life is improved with less impact on daily living and a return of sexual function. Nearly 90% of patients preferred TPS to IUC and the majority would “recommend TPS use to a friend" [9].

According to the World Health Organization, antibiotic-resistant organisms represent one of the biggest threats to global health [10]. Our pilot study suggests that replacing IUC with TPS results in the clearance of asymptomatic bacteriuria among previously colonized patients. If these findings are supported in larger data sets, TPS utilization could be a compelling new tool in the treatment of patients with otherwise untreatable multi-drug resistant organisms.

## Conclusions

The TPS restores natural filling and emptying of the bladder and contributes to the natural elimination of bacteria from the urinary tract. Its use is safe and well-tolerated by patients, and more research is needed among the larger population and in specific at-risk patient groups to determine its optimal role in the elimination of bacterial colonization.
